# Surgical fixation of displaced midshaft clavicle fractures: elastic intramedullary nailing versus precontoured plating

**DOI:** 10.1007/s10195-014-0298-7

**Published:** 2014-05-25

**Authors:** Nidhi Narsaria, Ashutosh K. Singh, G. R. Arun, R. R. S. Seth

**Affiliations:** 1 Mayo Institute of Medical Sciences, Barabanki, India; 2 Department of Orthopaedics, Mayo Institute of Medical Sciences, Barabanki, 226010 Uttar Pradesh India; 3 Department of Orthopedics, Sri Mokambika Institute of Medical Sciences, Kulasekharam, Tamilnadu India

**Keywords:** Displaced midshaft clavicle fractures, Elastic intramedullary nailing, Anatomical precontoured plating

## Abstract

**Background:**

This prospective comparative study was done to evaluate the effectiveness of implants of different design (titanium elastic intramedullary nail versus anatomical precontoured dynamic compression plate) in treatment of displaced midshaft clavicular fractures.

**Materials and methods:**

Sixty-six patients between 18 and 65 years of age were included in this study. They were randomized in two groups to be treated with either elastic intramedullary nail (EIN) or plate. Clinical and radiological assessments were performed at regular intervals. Outcomes and complications of both groups over 2 years of follow-up time were compared.

**Results:**

Length of incision, operation time, blood loss and duration of hospital stay were significantly less for the EIN group. American Shoulder and Elbow Surgeons (ASES) and Constant Shoulder scores were significantly higher (*p* < 0.05) in the plating group than the EIN group for the first 2 months but there was no significant difference found between the two groups regarding functional and radiological outcome at the 2-year follow-up. Significantly higher rates of refracture after implant removal (*p* = 0.045) in the plating group was observed. Infection and revision surgery rates were also higher in the plate group, but this difference was insignificant (*p* > 0.05).

**Conclusions:**

EIN is a safe, minimally invasive surgical technique with a lower complication rate, faster return to daily activities, excellent cosmetic and comparable functional results, and can be used as an equally effective alternative to plate fixation in displaced midshaft clavicle fractures.

**Level of evidence:**

Level 2.

## Introduction

Fractures of the clavicle account for 2.6–4 % of all adult fractures, 35 % of all injuries to the shoulder girdle, and 69–82 % of these fractures occur in the middle-third [1, 2]. Displacement occurs in about 73 % of all midshaft clavicle fractures [2]. The average age of patients sustaining a midshaft clavicular fracture is 33 years; 70 % of the patients are male [3]. A fall or a direct blow to the shoulder, giving an axial compressive force on the clavicle, is the most common trauma mechanism of injury for any clavicular fracture [4, 5]. Displaced midshaft fractures have traditionally been treated non-operatively because of early reports suggesting that clavicular nonunions were very rare and clavicular mal-union, being of radiographic interest only, was without clinical importance [6, 7]. However, recent studies have found higher rates of delayed union, nonunion, shoulder pain, and shoulder weakness and residual pain with non-operative treatment [8]. The indications for surgery include the need for earlier functional mobilization in the patient with an isolated injury, in addition to open fractures, floating shoulders and patients with polytrauma [9]. For operative treatment, the available methods of fixation are fixation with Kirschner wires, pins (Rush pin, Knowles pin, Rockwood pin), plates with screws and external fixation [10–12].

This prospective comparative study was designed to compare outcomes and complications of titanium elastic intramedullary nailing and anatomically precontoured plating in displaced midshaft clavicular fractures.

## Materials and methods

We conducted a prospective comparative study to compare outcomes and complications of closed displaced midshaft clavicular fractures treated with precontoured dynamic compression plates or with single titanium elastic intramedullary nails. Between July 2008 and June 2010, a total of 80 patients with closed displaced midshaft clavicular fractures were admitted in our hospital. Out of these 80 patients, 66 patients were included in this study. In this study, these patients were randomized according to inclusion and exclusion criteria into two equal groups of 33 patients, to be treated surgically with either a 3.5-mm precontoured dynamic compression plate (plate group) or with a single titanium elastic intramedullary nail fixation (EIN group).

### Inclusion criteria

- Age >16 and <65 years of age
- Duration <2 weeks
- Shortening of over 15 mm [8] and axial malalignment of over 30° with no cortical bone contact [13]
- Dislocation, defined as at least one shaft width difference in height between the fracture parts, regardless of the reduction [14].

Patients were excluded if they had fractures with marked comminution, duration of more than 4 weeks, open fractures, pre-existent morbidity of the ipsilateral arm, shoulder or hand, presence of neurovascular injury, and ipsilateral injuries.

The characteristics of the patients of both groups are shown in Table 1. Patients were randomized into two groups by the concealed envelope technique. The Robinson [1] classification system is the most valuable in terms of choosing therapy, as well as being of prognostic value for midshaft clavicular fractures. In this study we have included angulated midshaft clavicle (type 2A2) fractures and displaced midshaft clavicle (type 2B1) fractures. Type 2B2 fractures were not included in this study because these fractures were segmental and markedly comminuted. According to the Robinson classification system, 12 were type 2A2, 23 cases were of 2B1 type in plate group, 10 cases were type 2A2, 25 cases were type 2B1. The average age in the plating group was 40.2 ± 11.2 (range 18–64) years and in the elastic nailing group it was 38.9 ± 9.1 (range 20–62) years. Both groups showed no statistical difference in term of age (*p* = 0.82), gender (*p* = 0.64), and time from injury to operation (*p* = 0.62). Surgery was performed at a mean of 7.2 ± 3.2 days (range 1–14 days) of injury time in the plate group and at a mean of 6.9 ± 3.1 days (range 1–13 days) in patients in the EIN group, and there was no statistically significant difference (*p* = 0.62).

**Table 1:** Demographic profile of study

Characteristics	Precontoured plating group	Antegrade elastic nailing group	*p* value
Mean age (years)	40.2 ± 11.2 (18–64)	38.9 ± 9.1 (20–62)	0.82
Male:female	26:6	24:9	0.64
Right:left	20:12	18:15	0.80
Mean injury time (days)	7.2 ± 3.2 (1–14)	6.9 ± 3.1 (1–13)	0.62

**Fig. 1:**
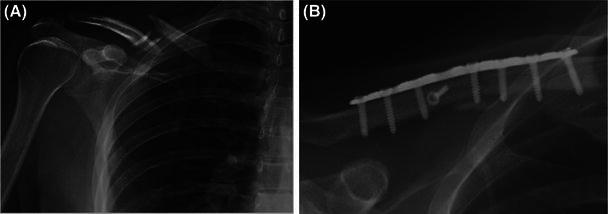
**a** Preoperative X-ray of 32-year-old female patient showing displaced midshaft clavicle fracture *right side*. **b** Immediate postoperative X-ray showing plate osteosynthesis with anatomical precontoured 3.5-mm dynamic compression plate

Internal fixation was done according to AO principles. After general anesthesia, the patient was positioned in the beach-chair position with a folded sheet under the affected shoulder. A transverse incision was made over the fracture site and dissection was carried out down to the fracture site, followed by careful subperiosteal dissection [15]. The fracture was reduced and held temporarily with bone clamps, and the plate was positioned on the anterior superior surface of the clavicle (Fig. 1a, b). Lots of different plates are being used nowadays in clavicle fracture fixation. In this study, we compared a precontoured 3.5-mm clavicular dynamic compression plate (Synthes) with EIN. Additional interfragmentary lag screws were used in cases of oblique fractures.

**Fig. 2:**
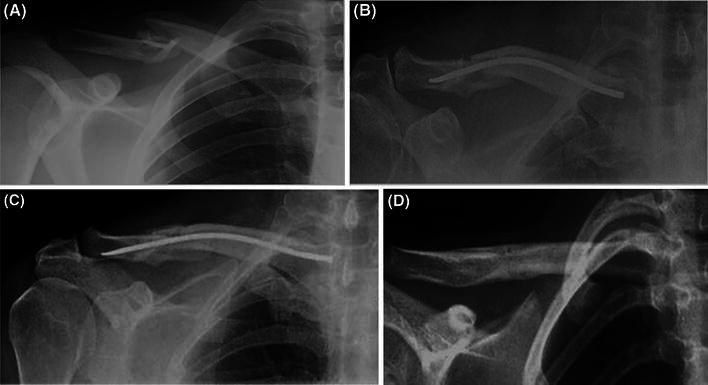
**a** Preoperative X-ray of 26-year-old male patient showing displaced midshaft clavicle fracture *right side*. **b** Fracture reduced and fixed with antegrade titanium elastic nail. **c** Postoperative X-ray at 12 weeks showed fracture uniting well with nail in situ. **d** Postoperative X-ray showing united fracture (elastic nail removed)

Elastic intramedullary nailing was done using the technique described first by Jubel et al. [16]. A small skin incision was made approximately 1 cm lateral to the sternoclavicular joint. The medullary cavity of the clavicle was opened using an awl pointed laterally and angled at about 30° to the coronal plane in line with the clavicle. Care was taken not to perforate the dorsal cortex in order to avoid major complications. Single elastic nails of different diameters varying from 2 to 3.5 mm, were used, depending on the width of the bone. To obtain the exact position of the titanium elastic nail (TEN), fluoroscopy with true perpendicular views was used. Closed reduction was done under an image intensifier, and provisionally fixed with two percutaneously pointed reduction clamps. In 15 cases of the EIN group, close reduction of the fracture site could not be done, so an additional small incision was made above the fracture site for direct manipulation of the main fragments before the nail was introduced into the lateral fragment and the fracture was compressed. Care was taken to avoid perforation of the dorsolateral cortex of the lateral clavicle. The TEN was cut as short as possible at the medial end (Fig. 2a–c). In all cases, elastic nails of the same make (Synthes) were used.

In both groups, arm sling support was given to all the patients for 2 weeks postoperatively. Early mobilization was started if pain permitted. Patients were encouraged to resume their normal daily activities after a 4-week postoperative period.

Operative time, length of incision, hospital stay, blood loss (calculated by the difference in the weights of the sponges pre- and postoperatively and adding volumes of suction loss), and pain visual analogue scale (0: none to 10: severe) on the first post-operative day were recorded for each patient. In follow-up visits, all patients were evaluated clinically at 1st, 2nd, 4th, 6th, 12th, 18th and 24th month to assess outcomes of fracture fixation in both groups, like fracture union time, union rate, shoulder and arm function. Shoulder function was evaluated according to the American Shoulder and Elbow Surgeons (ASES) score and Constant score, (both are 100-point scoring systems) [17]. These scoring systems combine assessments of subjective symptoms and objective findings. In the Constant scoring system, the overall grading is excellent if the total score ranges from 90 to 100, good for 80–89, fair for 70–79, and poor if the scores are 69 or less.

Complications were recorded and compared between both groups. Non-union was defined as an unsuccessful healing of the bone after 6 months, clinically manifesting as pain at the fracture site and radiologically as a visible gap between the fracture parts. Deep infection was defined as infection requiring implant removal. Refracture was defined as a fracture of the clavicle within 3 months of implant removal without any history of retrauma.

Student’s *t*-test was used to analyze the difference of means for different parameters. Mean, standard deviation and standard error of mean for the variables were also calculated. The test was referenced for a two-tailed *p* value, and a 95 % confidence interval was constructed around a sensitivity proportion using a normal approximation method. Statistical analyses were performed using SPSS software. A value of <0.05 was considered statistically significant.

## Results

In this study, during a 2-year period from July 2008 to June 2010, 66 patients with displaced midshaft clavicle fractures were included as per inclusion criteria and underwent surgical fixation. In the EIN group, closed fracture reduction and internal fixation was done in 14 cases (42.42 %), and open reduction was required in the remaining 18 patients (56.25 %). There was significant difference in both groups (less in EIN group) regarding mean operative time (*p* = 0.041), blood loss (*p* = 0.004) and length of hospital stay (*p* = 0.032) as shown in Table 2. The average bone union time was shorter in the EIN group (6.1 months ± 1.8; range 2.5–8 months) than in the plating group (7.4 months ± 2.7; range 3–11 months) but this difference was insignificant (*p* = 0.68).

**Table 2:** Comparison of perioperative measures and outcomes of both groups

Outcome	Precontoured plating group	Antegrade elastic nailing group	*p* value
Surgery time (min)	58.4 (50–82)	40.2 (28–55)	0.041
Length of incision (cm)	10.2 (8.5–12)	4.5 (3–5.5)	0.008
Pain (visual analogue scale)	4 (2–6)	3 (2–9)	0.18
Hospital stay (days)	2.8 (1–4)	1.4 (1–2)	0.032
Average blood loss (ml)	130.8 (80–164)	70.0 (35–94)	0.004
Union rate	32 (100 %)	32 (96.96 %)	0.42
Union time (months)	7.4 (3–11)	6.1 (2.5–8)	0.68

Two cases in the plate group and one case in the EIN group developed superficial infection (*p* = 0.62) but infection was controlled by oral antibiotics in all three cases. There was no deep infection in any case of both groups. Nonunion occurred in one case in the EIN group, while in the plating group, all fractures united (*p* = 0.84) (Table 3).

**Table 3:** Comparison of complications of both groups

Complications	Precontoured plating group (%)	Antegrade elastic nailing group (%)	*p* value
Infection	2 (6.25)	1 (3.03)	0.62
Implant failure	0 (0.0)	1 (3.03)	0.41
Wound dehiscence	3 (9.37)	0 (0.0)	0.046
Hypertrophic scar	4 (12.50)	0 (0.0)	0.04
Refracture after implant removal	3 (9.37)	0 (0.0)	0.046
Nonunion	0 (0.0)	1 (3.03)	0.41
Major revision surgeries	2 (6.25)	1 (3.03)	0.62

No implant failure occurred in the plate group while one implant failure was seen in the EIN group (3.03 %) (*p* = 0.41), which occurred within three months of the primary surgical procedure. Open reduction and plating with autogenous bone grafting in this case finally resulted in bone union. Three refractures (9.37 %) were observed in the plate group after removal of the implant without any history of fresh trauma while no such complication was seen in the EIN group (*p* = 0.046). All refractures occurred within 1 month after plate removal. The average age of the patients having refractures after plate removal was 37.9 years. Of these three refractures, one was treated conservatively and plating was done in two cases, leading to uneventful healing. Hypertrophic scar formation was observed in four cases in the plating group, none in the EIN group (*p* = 0.04); wound dehiscence was seen in three cases in the plating group and none in the EIN group (*p* = 0.046).

In the EIN group, elastic nails were removed in all cases. In the plate group 20 patients (total of 32 patients) underwent implant removal. In the EIN group the nail was removed at an average time of 6.2 ± 1.6 months (range 4–9 months). Plates were removed at an average time of 15.4 ± 2.2 months (range 11–20 months) (*p* = 0.02).

ASES and Constant Shoulder scores were assessed at every follow-up visit and the 2-month postoperative follow-up visit showed significantly higher Constant scores of 74.1 ± 8.2 in the plating group than in the EIN group (60.1 ± 10.2) (*p* = 0.04). The final scores at the 24-month follow-up visit showed no significant difference between two groups, as shown in Table 4 (*p* > 0.05).

**Table 4:** Comparison of ASES scores and Constant scores [15] of both groups

Scores	Precontoured plating group		Antegrade elastic nailing group		*p* value
	Mean	Standard deviation	Mean	Standard deviation	
ASES score—subjective					
Pain	9.1	0.3	9.3	0.2	0.42
Activities	28.4	0.8	30.3	0.6	0.62
ASES score—objective					
Range of motion	38.8	0.8	35.6	0.7	0.81
Strength	19.2	0.4	20.5	0.2	0.64
Total ASES score	99.4	0.6	96.8	3.0	0.39
Constant score—subjective	34.2	1.2	30.3	1.8	0.81
Constant score—objective	62.7	2.4	60.6	2.9	0.74
Total Constant score	96.2	2.6	94.6	3.2	0.83

## Discussion

The best treatment strategy for displaced midshaft clavicle fractures remains a topic of debate. Conservative management of these fractures results in an approximately 5 % nonunion rate [4]. While non-operative management remains the mainstay of treatment for most midshaft clavicle fractures, the indications for surgery may be expanding. Recent studies have showed a poorer outcome in cases of displaced midshaft clavicle fractures that were treated non-operatively [8, 18, 19] in comparison to surgically treated patients [16, 20, 21]. Three types of fixation are available for middle-third clavicle fractures: intramedullary devices, plates, and external fixators. Intramedullary fixation can be done by smooth or threaded K- wires, Steinman pins, Knowles pins, Hagie pins, Rush pins or cannulated screws [22–24]. Plate fixation can be done with a 3.5-mm dynamic compression plate (DCP), low-contact dynamic compression plates, reconstruction plates or locking compression plates with at least three screws (six cortices) in both the medial and lateral fragment each, and an interfragmentary lag screw whenever the fracture pattern allows it. Plating of acute clavicle fractures is advocated as the preferred fixation method by many authors [15, 25, 26]. Biomechanically, plate fixation is superior to intramedullary fixation because it better resists the bending and torsional forces that occur during elevation of the upper extremity above shoulder level [27]. Patients treated with plate fixation can be allowed full range of motion once their soft tissues have healed. Disadvantages of plate fixation include the necessity for increased exposure and soft-tissue stripping, increased risk of damage to the supraclavicular nerve, slightly higher infection rates, and the risk of refracture after plate removal [7]. Currently, open reduction and internal fixation with a 3.5-mm dynamic compression plate [28, 29] is the standard method; however, intramedullary fixation [16, 30] is an equally effective alternative. In this study, both methods of fixation were compared in terms of outcomes and complications.

In our study, functional shoulder scores were significantly higher for the plating group than the EIN group in the first 12 weeks, but at the 12-month follow-up visit, there was no significant difference observed between the two groups in terms of shoulder scores. In this study, in the plating group, rates of refracture (9.37 %), major revision surgery (6.25 %) and implant failure (3.03 %) were comparable to other studies. The Canadian Orthopaedic Trauma Society reported one (1.6 %) case of early mechanical failure [5]. Böstman et al. [31] studied 103 patients treated with open reduction and internal fixation using plates; among those patients, 43 % had complications; 15 %, major complications; 14 % required re-operation and there was an implant failure rate of 14.6 %. Chen et al. [32] reported a 7.1 % implant failure rate. Liu et al. [33] compared titanium elastic nail and reconstruction plate fixation in displaced midshaft clavicle fractures and found no significant difference between intramedullary and plate fixation after 18 months in terms of functional outcome (DASH score *p* = 0.42, Constant score *p* = 0.17) and complications. They reported an implant failure rate of 8.5 %. In our study, the refracture rate was significantly greater in the plate group than in the EIN group. Wijdick et al. [14] analysed retrospectively 90 patients with displaced mid clavicle fractures treated with plate fixation or EIN. Complications were evaluated in both treatment groups and subsequently compared. Three refractures (7.0 %) were observed in the plate group after removal of the implant against none in the EIN group (*p* = 0.105). All refractures occurred within 2 months after removal of the implant. Poigenfurst et al. [15] followed 122 patients after plating of displaced clavicle fractures. There were four refractures after plate removal. The reason behind this higher refracture rate after implant removal in the plating group is that plate fixation provides a rigid fixation leading to primary bone healing: that’s why, after plate removal, the mechanical strength of the healed fracture site is reduced, explaining higher refracture rates. Along with this, screw holes may act as focal points for stress, leading to refractures. Secondary bone healing occurs in cases of fractures treated with EINs so the refracture rate after removal of the implant is lower in these cases. For plate fixation a larger incision is required, leading to a higher risk of infection and lesser cosmetic satisfaction but in our study no significant differences in infection rates between the two groups were found.

Ferran et al. [34] compared Rockwood pin fixation (17 cases) and low contact dynamic compression plate (LCDCP; 15 cases) in displaced midshaft clavicle fractures and found no significant difference after 12 months in functional outcome (Constant score *p* = 0.37). Complications occurred in 12 % of the intramedullary fixation group and in 40 % of the plate fixation group. Bohme et al. [35] reported the same conclusions in their study comparing plating, intramedullary fixation and conservative treatment in displaced midshaft clavicle fractures. Thyagarajan et al. [36] retrospectively evaluated 51 patients (three groups, each had 17 patients) with midshaft clavicle fractures. Group 1 underwent intramedullary stabilization using clavicle pins. Group 2 underwent open reduction and internal fixation using plates and group 3 underwent non-operative treatment with a sling. In group 2, two (12 %) patients had prominent hardware causing discomfort, and they underwent removal of hardware 12 months following the fixation. After implant removal results were satisfactory and there was no incidence of refracture.

In a retrospective study done by Wu et al. [37], comparison between plating and intramedullary nailing for the treatment of clavicular nonunion showed an 18.2 % nonunion rate with plating compared with 11.1 % for nailing, the difference being attributed to the nailʼs resistance to compressive stresses. The authors concluded that plating provides better rotational stability. Several other studies have found intramedullary fixation to be equally effective as plating, especially for the treatment of nonunion [38, 39]. Refracture after implant removal and major revision surgery just tended to prevail more often after plate fixation, while implant failure was more common in EIN groups. Major revision procedures were done in EIN groups due to implant failure, while in plating groups it was due to refracture after implant removal. Minor revision surgeries were common in EIN groups for problems like medial protrusion causing irritation or skin perforation. Major complications described in the literature for other modes of intramedullary fixation of clavicle fractures (Kirschner wire, Rush pin etc.), like injury to neurovascular structures and implant migration into the chest cavity [40, 41] were not observed in our study. No such complication has been described in the literature using TENs in clavicle fractures [16]. Implant removal in the plating group needed another surgery done under general anesthesia, and a large-sized incision was made, while in the EIN group the nail was removed as an outdoor procedure under local anesthesia and a small incision over the tip of the nail was made. This was another advantage of intramedullary flexible nailing over plating.

The primary limitation of our study was that it was a small prospective comparative study including a small number of patients and done at a single center. Larger randomized controlled trials are needed to further evaluate outcomes and complications of precontoured plates and EIN in displaced midshaft clavicle fractures. Still, we can conclude from our study that both precontoured plating and intramedullary flexible nailing are equally effective alternatives for surgical fixation of displaced midshaft clavicular fractures. Antegrade flexible intramedullary nailing techniques have advantages like less soft tissue injury, shorter operating time and hospital stay, less blood loss, more cosmetic satisfaction and minor surgery needed to remove the implant. EIN is a safe, minimally invasive surgical technique with a lower complication rate, faster return to daily activities, excellent cosmetic and comparable functional results, which can be regard as an alternative to plate fixation of displaced midshaft clavicular fractures.
